# Mechanisms of Phase Transformation and Creating Mechanical Strength in a Sustainable Calcium Carbonate Cement

**DOI:** 10.3390/ma13163582

**Published:** 2020-08-13

**Authors:** Jesús Rodríguez-Sánchez, Teresa Liberto, Catherine Barentin, Dag Kristian Dysthe

**Affiliations:** 1 Physics of Geological Processes (PGP), The NJORD Centre, Department of Physics, University of Oslo, P.O. Box 1048 Blindern, 0316 Oslo, Norway; j.rodriguez-sanchez@sheffield.ac.uk; 2 Department of Materials Science and Engineering, University of Sheffield, Sheffield S10 2TN, UK; 3 Building Physics and Construction Ecology, Faculty of Civil Engineering, Institute of Materials Technology, Vienna University of Technology, 1030 Vienna, Austria; teresa.liberto@tuwien.ac.at; 4 Institut Lumière Matière, Université Claude Bernard Lyon 1, CNRS, F-69622 Villeurbanne, France; catherine.barentin@univ-lyon1.fr; 5 Institut Universitaire de France, 75231 Paris, France

**Keywords:** phase transformation, hardening, colloidal suspension, calcium carbonate, cement

## Abstract

Calcium carbonate cements have been synthesized by mixing amorphous calcium carbonate and vaterite powders with water to form a cement paste and study how mechanical strength is created during the setting reaction. In-situ X-ray diffraction (XRD) was used to monitor the transformation of amorphous calcium carbonate (ACC) and vaterite phases into calcite and a rotational rheometer was used to monitor the strength evolution. There are two characteristic timescales of the strengthening of the cement paste. The short timescale of the order 1 h is controlled by smoothening of the vaterite grains, allowing closer and therefore adhesive contacts between the grains. The long timescale of the order 10–50 h is controlled by the phase transformation of vaterite into calcite. This transformation is, unlike in previous studies using stirred reactors, found to be mainly controlled by diffusion in the liquid phase. The evolution of shear strength with solid volume fraction is best explained by a fractal model of the paste structure.

## 1. Introduction

Due to the carbon footprint of ordinary Portland cement (OPC), there has been an increased interest in alternative hydraulic binders [1,2,3,4]. Inspired by the outstanding mechanical properties of limestone, which is mainly composed of calcium carbonate (CaCO_3_), alternative production paths to form calcium carbonate binders have been investigated [1,5,6], the most famous being the Calera carbonation process [7]. Another path to prepare pure CaCO_3_ cements is of special interest as a model system: Combes and co-workers [5,6] mixed water with two metastable CaCO_3_ phases. One of them is the highly reactive amorphous calcium carbonate (ACC), while the other is one of the metastable crystalline phases, either vaterite (V) or aragonite (Ar). The polymorphs ACC and V or Ar recrystallize into the most stable polymorph, calcite, during the setting of the cement. 

However, the mechanical properties still remain poorly understood despite their chemical similarities with limestone. These properties develop during the process of preparing the initial powders, mixing the paste, setting reactions at constant liquid fraction and during drying. In this paper, we focus on the properties of a calcium carbonate paste while it is transforming from ACC and vaterite to calcite, at constant liquid fraction. We combine measurements of the phase transformation with rheological properties and interpret this in terms of mechanisms of chemical, physical and structural changes. This double approach allows us to link the microscopic properties with the mechanical properties of the paste.

Recrystallization of CaCO_3_ polymorphs has received much attention in recent years [8,9,10,11,12,13,14,15,16] due to the importance of CaCO_3_ in industry [17], climate [18], biomineralization [19] and geology [20]. This large body of knowledge on recrystallization mechanisms has yet to be put into the context of CaCO_3_ cement setting. In this paper, we compare CaCO_3_ setting reactions with the transformation mechanisms in other settings and then we compare this to the development of elastic properties of the CaCO_3_ cement paste during setting. 

Rheological measurements are efficient tools to investigate the structure of concentrated pastes and the interaction between particles [21]. In particular, the range of solid fractions for which elastic or yield stress behavior allows to determine the sign of the interparticle forces. In the case of attractive colloidal gels, the pastes are composed by a continuous percolating network of aggregated particles characterized by a fractal dimension. Models of mechanical deformation of such networks link the structure, the solid fraction and the elastic modulus or yield stress [22,23,24].

Most studies of rheology of concentrated particle suspensions (pastes) have been performed on timescales where the particles do not react, although some studies of setting cement pastes have been performed [25]. In the present system, however, the initial particles (ACC and vaterite) of the paste disappear completely while new calcite particles are forming. The properties of hardened CaCO_3_ cements are a result of the (re)crystallization that the paste undergoes when the solid reagents are mixed with water. It is therefore of crucial importance to correlate these phase transformations with the evolution of the viscoelastic properties of the paste during maturation to unravel the strengthening mechanisms and their kinetics [26,27].

In the present study, sustainable pure calcium carbonate cements have been synthesized following “Combes’ method” [6]. In-situ X-ray diffraction (XRD) scans and rheological measurements have been carried out to investigate the interplay between (i) the transformation kinetics of each phase (ACC, vaterite and calcite) of the paste and (ii) the development of mechanical properties during setting.

## 2. Materials and Methods 

CaCO_3_ cement samples with diverse mixture design have been prepared. Both the composition and the (re)crystallization kinetics of each CaCO_3_ phase (ACC, vaterite and calcite) along setting and hardening processes have been studied by in-situ time-lapse XRD. Then, the effect of ACC and vaterite (re)crystallization on the viscoelastic properties of the pastes have been quantified by rheological measurements.

### 2.1. Synthesis of CaCO_3_ Polymorphs

The preparation of CaCO_3_ cements requires the synthesis of different calcium carbonate polymorphs in order to design the initial cement mixture [6]. Accordingly, both ACC and vaterite phases were precipitated by mixing calcium chloride (CaCl_2_·6H_2_O, Sigma Aldrich, Saint Louis, Missouri, MO, USA) and sodium carbonate (Na_2_CO_3_, Sigma Aldrich, Saint Louis, Missouri, MO, USA) equimolar (0.5 M) solutions, prepared with ultrapure Milli-Q water (Millipore Corporation, Burlington, Massachusetts, MA, USA) (18.2 MΩ cm), at room temperature using a magnetic stirrer (Cole-Parmer, Vernon Hills, Illinois, IL, USA) set at 400 rpm. Following Ogino et al. [8], we used a reaction time of 10 s for ACC and 30 min for vaterite. To wash off any dissolved sodium chloride (NaCl), the CaCO_3_ precipitates were rinsed with deionized water and ethanol (Sigma Aldrich, Saint Louis, Missouri, MO, USA), using a filtering kit (Millipore, Burlington, Massachusetts, MA, USA) and 0.5 µm pore-size filter papers (Millipore, Burlington, Massachusetts, MA, USA). Subsequently, the CaCO_3_ precipitates were flushed with liquid N_2_ (Air Liquide, Mjøndalen, Norway) to stop the recrystallization of these metastable phases. The frozen precipitates were lyophilized (Alpha 1-4 LD plus, Martin Christ, Osterode am Harz, Germany) for 48 h at 0.05 mbars and −50 °C and stored in a freezer (−22 °C) (Samsung, Seul, South Korea) with additional silica powder (Sigma Aldrich, Saint Louis, Missouri, MO, USA) bags to keep them as dry as possible [13] prior to cement manufacturing.

The precipitates were scanned by XRD (Bruker D8 Discover X-ray diffractometer, Billerica, Massachusetts, MA, USA) to identify the crystalline phases present using Cu Kα radiation (30 mA and 40 kV) and recording 2*θ* angles from 20° to 50° at a 0.04 s^−1^ sweep rate. Additionally, they were imaged with scanning electron microscopy (SEM) (Hitachi SU5000 Field-Emission; 1 kV acceleration voltage and 3.0 mm working distance, Chiyoda, Japan).

### 2.2. In-Situ XRD Analysis during Setting and Hardening

To quantify the composition and the (re)crystallization kinetics of CaCO_3_ cement pastes during the setting and hardening processes, we have used in-situ time-lapse XRD (Bruker D8 A25 X-ray diffractometer). Five CaCO_3_ cement pastes were made up of mixtures of ACC and vaterite powders in different weight ratios (wt.%): 1:0, 1:1, 1:2, 1:3 and 0:1, with a solid volume concentration, Φ, equal to 46% (which is equivalent to a 0.43 water to solid ratio, W/S). This concentration was deduced from the total powder weight and ACC, vaterite and water densities. Table 1 summarizes the CaCO_3_ cement compositions and their nomenclature. Just after mixing the powders with the liquid phase, the resulting pastes were viscous and easily moldable for several minutes, and they were molded into the sample holder (disk of 25 mm diameter, 2 mm depth), firmly tamped and covered with a plastic film immediately after mixing with water to avoid drying.

XRD patterns were recorded every 5 min for a maximum of 60 h using Mo Kα radiation (48 mA and 48 kV) and recording 2θ angles from 10° to 17° at a sweep rate of 0.035 s^−1^. Since the mass of a crystalline phase, m_i_, is proportional to the area, A_i_, under its diffraction peak, the net area under the main vaterite and calcite diffraction peaks (located at 12.35° and 13.39° respectively, within the range scanned) were calculated using the software Diffrac.Suite EVA (Bruker AXS, Billerica, Massachusetts, MA, USA) for each scan. The mass fractions of each phase, X_i_, can be calculated from A_i_ based on two assumptions: the initial vaterite mass fraction is the same as prepared in the mixture proportions and in the final scan the cement is pure calcite minus the unreacted vaterite. Then, the mass fractions of vaterite, calcite and ACC can be calculated by $X_{V}\left(t\right)=X_{V}\left(t_{0}\right)\cdot \frac{A_{V}\left(t\right)}{A_{V}\left(t_{0}\right)}$, $X_{C}\left(t\right)=\frac{A_{C}\left(t\right)}{A_{C}\left(t_{f}\right)}\left(1-X_{v}\left(t_{f}\right)\right)$ and $X_{\mathrm{ACC}}=1-X_{C}-X_{V}$, where t represents the time, t_0_ the initial time, t_f_ the final time and X_V_ (t_0_) the starting vaterite mass fraction which is determined by the mixture design, i.e., either 0, 0.5, 0.66, 0.75 or 1, for the ACC:V 1:0, 1:1, 1:2, 1:3 and 0:1 pastes, respectively. 

### 2.3. Rheological Characterization

#### 2.3.1. Sample Preparation

CaCO_3_-cement colloidal suspensions were prepared by dispersion of ACC and vaterite powdered mixtures in distilled water. Three different mixtures were made up by mixing ACC and vaterite powders in different weight ratios (wt.%): 1:1, 1:2 and 1:3, respectively (see Table 1). The selected solid volume concentration, Φ, was 36% (which is equivalent to a 0.65 water to solid ratio, W/S) based on preliminary tests to determine the suitable concentration range for rheological measurements (storage modulus, G′, > loss modulus, G″). We could not study higher concentrations, Φ, due to some limitations of the rheometer and in particular due to its maximal torque. This concentration was deduced from the total powder weight and ACC and vaterite densities within the paste. The mixing of the solid and the liquid phase was carried out in a vortex stirrer (Ultra Turrax TD300, IKA, Staufen, Germany) at 5800 rpm for 20 s. This time was checked to be long enough to assure a homogeneous and easily moldable paste without modifying its viscoelastic properties. After the mixing step, the samples were immediately transferred into the rheometer geometry and studied.

#### 2.3.2. Measurements Protocols 

The viscoelastic properties of the cement pastes (ACC:V mixtures) were studied using a stress-controlled rotational rheometer (Anton Paar MCR 301, Graz, Austria) in oscillating mode. The measurements were carried out using a plate–plate geometry (36 and 64 mm, upper and lower diameters, respectively) at room temperature. The gap between the two plates was fixed to 1 or 2 mm. Both plates were covered with sand-paper P320 (roughness 46 μm) to prevent slippage [28]. This was checked using diffusing-wave spectroscopy (DWS) across the rheometer gap [29]. In addition, a moisture chamber was used to assure a water-saturated atmosphere and thus dispel any paste-drying effect during the data acquisition.

From the measurements, the storage modulus, *G*′, and the loss modulus, *G*″, were extracted as the characterizing parameters of the viscoelastic behavior. Those are defined by [21]:

(1) $$G^{*}=G^{\prime }+iG^{\prime \prime }$$

where G* is the complex modulus, being the ratio between the complex stress, σ*, and the complex strain, γ*:

(2) $$\sigma^{*}\left(t\right)=G^{*}\cdot \gamma^{*}\left(t\right)$$

Two different experimental protocols were used. The first one aimed at studying the evolution of the ACC and vaterite transformation reaction by measuring the time evolution of the storage modulus *G*′(*t*) during 10 h. The second one aimed at characterizing the elastic properties of the CaCO_3_ cement paste just after its preparation.

##### Viscoelastic Measurements during Setting Reaction: Aging Experiment

To quantify the influence of ACC and vaterite recrystallization into calcite on the macroscopic mechanical properties of pastes, a constant oscillatory deformation, *γ* = 0.003%, was applied to the pastes at the imposed frequency, f = 10 Hz, for several hours. This experiment allows measuring the time evolution of the viscoelastic moduli (*G*′, *G*″) during the setting reaction in the linear regime. Indeed, the values of strain and frequency (*γ* = 0.003%, f = 10 Hz) were chosen small enough to ensure measurements within the linear elastic regime for all the tested pastes, and thus no perturbation of the setting and hardening dynamics of the samples. The measurements started immediately after mixing the solid and the liquid phase, transferring the pastes into the plate–plate geometry and sealing the setup with the moisture chamber.

##### Viscoelastic Properties and Yield Stress of Initial Pastes

The pastes were prepared as described in Section 2.3.1 and transferred into the plate–plate setup sealed with the moisture chamber. In the aim to characterize the viscoelasticity and the yield stress of fresh paste, increasing stresses are imposed and resulting strains are measured. At low stresses and low strains, the storage modulus, *G*′, is constant, so the linear storage modulus, *G′_lin_*, can be estimated precisely. At larger strains, the pastes eventually yield and become liquid-like, causing the storage modulus, *G*′, to break down. The end of the linear regime, i.e., the onset of plasticity, was characterized by the critical strain, *γ_CR_*, which corresponds to the value of *γ* for which the storage modulus was 10–15% lower than the value in the elastic regime, *G′_lin_* [28].

## 3. Results and Discussion

In this section, we present the XRD measurements corresponding to the recrystallization of calcium carbonate polymorphs from ACC and vaterite to the final calcite cement. Subsequently, the time evolution of the paste rheology is presented. Two different timescales of transformation by recrystallization, particle interactions and strength development are identified and modelled.

### 3.1. Characterization of CaCO_3_ Precipitates

**Figure 1:**
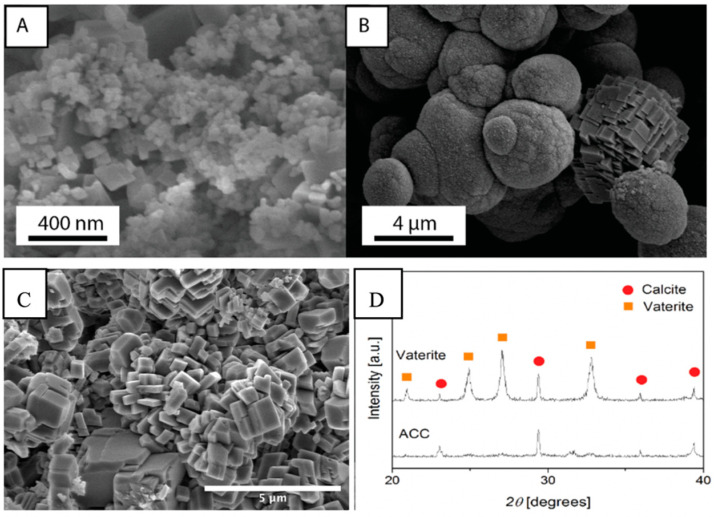
Scanning electron microscopy (SEM) micrographs of synthesized calcium carbonate powders in the form of (**A**) Amorphous Calcium Carbonate (ACC) phase and (**B**) vaterite phase with one calcite grain. (**C**) Calcite cement after recrystallization of ACC:V 1:3 mixture. (**D**) X-ray diffraction (XRD) patterns of lyophilized ACC and vaterite powders.

To check the validity of the synthesis protocols described for both ACC and vaterite, the dried precipitates used as starting materials for the different cement compositions are investigated by SEM and XRD. The SEM analysis indicates that ACC precipitates (Figure 1A) consist of equidimensional spherical nanoparticles (< 50 nm in diameter) accompanied by rhombohedral crystals of about 50–100 nm. These are interpreted to be calcite and confirmed by XRD analysis (Figure 1D). Vaterite precipitates (Figure 1B) consist of spherical grains of 2–5 µm in diameter also accompanied by rhombohedral grains (4–5 µm), which are calcite (Figure 1C) according to the XRD scans (Figure 1D). Magnification of vaterite grains (Figure 6) suggests that they consist of ~100 nm spheres. After (re)crystallization of ACC and V cement mixtures, calcite is the only crystalline phase present (Figure 1C, ACC:V 1:3 as an example), with grains well faceted and grain boundaries well developed. Typical grain size varies from 0.3 to 1 µm, with a few larger and smaller. An analysis of the XRD patterns using the software Diffrac. Suite TOPAS (Bruker AXS, Billerica, Massachusetts, MA, USA) derived a composition of 86% ACC and 14% calcite for ACC precipitates, while 93% vaterite and 7% calcite for vaterite precipitates, hence, some calcite will always be present in the initial cement powders.

### 3.2. Phase Transformation during Setting and Hardening

#### 3.2.1. Measurement of Time Evolution of Different Phases

**Figure 2:**
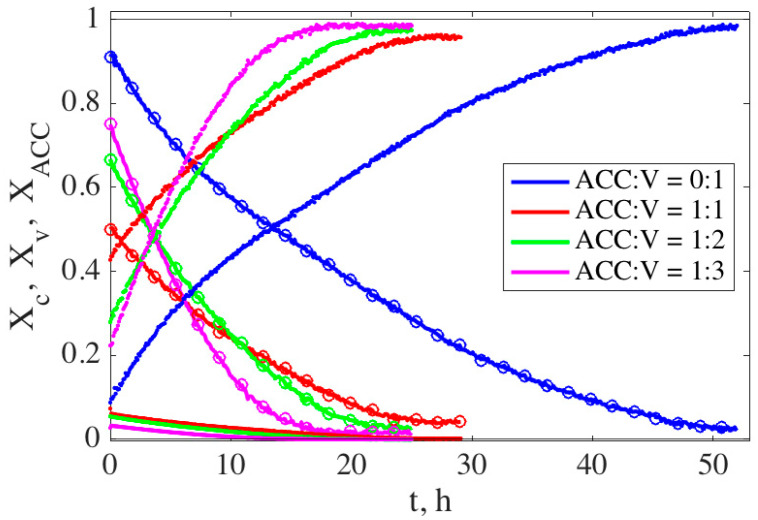
Mass fraction evolution of calcite, X_C_ (increasing with time), vaterite, X_V_ (circles), and ACC, X_ACC_ (always below 0.1), with time for four different CaCO_3_ cement compositions.

The time evolution of the cement compositions during the setting reaction has been followed by in-situ time-resolved XRD. Figure 2 displays the calculated molar fractions, Xi, of the three calcium carbonate polymorphs (ACC, vaterite and calcite) that coexist during the process for three different CaCO_3_ cement compositions (ACC:V 1:1, 1:2 and 1:3) as well as for pure ACC (ACC:V 1:0) and pure vaterite (ACC:V 0:1) pastes as references. It visually shows how the two starting phases—ACC and vaterite—are dissolving, while the third one—calcite—is growing at the expense of the other two. One also observes that the rate of transformation slows down with time. 

For the three cement compositions including both ACC and vaterite, the results show a clear trend: higher initial vaterite concentration leads to faster growth rates and a faster recrystallization process. However, pure vaterite (ACC:V 0:1 wt.%) shows the slowest transformation kinetics, probably due to its lower free-energy difference, Δ*G* (6.2 kJ·mol^−1^ [30]), with respect to calcite in comparison with ACC (15.0 kJ·mol^−1^ [14]). The ACC dissolves very quickly in all cases.

#### 3.2.2. Analysis of Phase Transformation Kinetics: Rate-Limiting Step

The transformation of ACC through vaterite to calcite is dominated by the sequential process of (1) dissolution of vaterite, (2) diffusion of CaCO_3_ from the dissolution site to a growth site and then (3) growth of calcite. If one of these three processes is significantly slower than the other ones, it is called the ‘rate-limiting step’ Before proceeding with our analysis is worthwhile to specify that the aim of this paper is not to study in details the complex transformation of ACC into calcite—this aspect has been extensively studied in stirred reactors [8,11]—but to elucidate the effective behavior of this transformation in a porous system to obtain a cement. The dissolution (growth) flux at a solid–liquid interface is proportional to the thermodynamic driving force times a rate constant. The overall rate of dissolution (growth) in the system is therefore proportional to the surface area of the dissolving (growing) crystals. Since the vaterite surface area is initially large and diminishes, vaterite dissolution rate control predicts that the transformations will start out at a high rate and slow down. Since calcite surface area is initially small and growing, calcite growth rate control predicts that the transformations will start out at a low rate and accelerate. Since surface area of the crystals scales with the mass to the power of 2/3, we may write:

(3) $$\frac{dm_{c}}{dt}\propto \;m_{c}^{2/3}\;\Rightarrow \;X_{C}\propto {\left(\frac{t}{t_{f}}\right)}^{3}$$

for calcite growth control and

(4) $$\frac{dm_{v}}{dt}\propto -m_{v}^{2/3}\;\Rightarrow \;X_{V}\propto {\left(1-\frac{t}{t_{f}}\right)}^{3}\Rightarrow \;X_{C}\propto 1-{\left(1-\frac{t}{t_{f}}\right)}^{3}$$

for vaterite dissolution control. The final time, *t_f_*, is the time when the mass fraction reaches the final plateau value.

If diffusion from the dissolution site to the growth site is rate-limiting, one has to make some assumption about what the diffusion distance is. Assuming that the size of the calcite grains is the pertinent length scale, the diffusion-limited model of the calcite mass fraction can be expressed as (see Supplementary Material, S1. Growth and dissolution rates):

(5) $$X_{C}\propto {\left(\frac{t}{t_{f}}\right)}^{3/5}.$$

**Figure 3:**
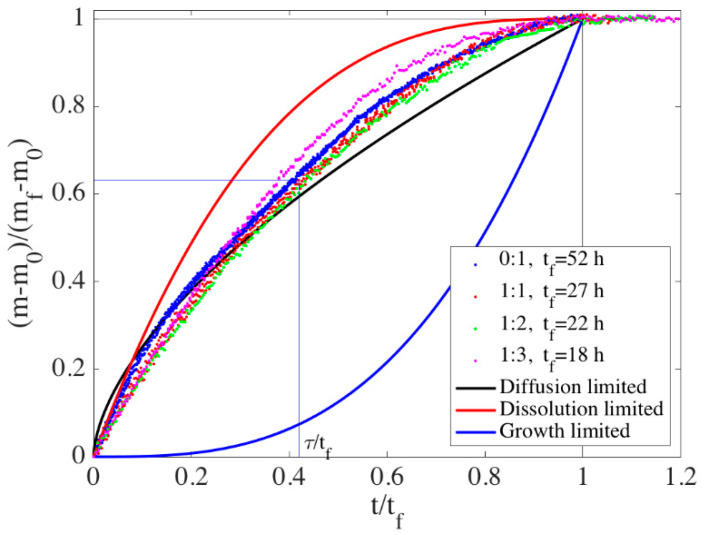
Mass evolution with normalized time for four different CaCO_3_ cement compositions. The solid line shows the predicted evolution from models with diffusion, vaterite dissolution and calcite growth as the rate-limiting steps.

In Figure 3, we have rescaled the time evolution of the vaterite and calcite mass by the initial and final mass and rescaled the time by the final time, *t_f_*. This rescaling collapses all the data onto a single curve that can be compared to the three models of rate-limiting step: diffusion, dissolution and growth (see Supplementary Material, S.2 Analysis of phase transformation data for more details). It is immediately clear that the transformation of ACC–vaterite–calcite in a cement paste is not limited by the calcite growth. In previous studies, using stirred liquid reactors where 1 M solutions of CaCl_2_ and Na_2_CO_3_ are mixed, the overall transformation from ACC to calcite was calcite growth-rate-controlled [8,11] (like the blue line in Figure 3). There are two main reasons why our result is so radically different: (1) cements and porous media are not stirred and (2) heterogeneous nucleation of calcite in a cement lowers the nucleation barrier and increases the surface area for growth. In a stirred liquid reactor, the main point is to avoid diffusion limitation of the rates so that pure reaction rates may be measured. However, nucleation seems to be a limit to the general validity of the stirred liquid reactor results since independent measurements of the dissolution and growth rates of vaterite and calcite find that the calcite rate constant is 2–10 times larger than the vaterite dissolution constant [31,32,33]. In a cement or porous media on the other hand, there will be some influence of diffusion unless the growing phase is nucleated everywhere. The data presented in Figure 3 fall somewhere between the simple diffusion-limited model—with calcite radius as the diffusion length scale—and the dissolution-limited model. 

To compare the timescale of phase transformations with the timescales of rheology, we use a standard definition of the relaxation time, τ: ln[(*m*_0_ − *m*)/(*m_f_* − *m*_0_)] = ln[X] = *t*/τ, that is τ~0.4 *t_f_*. The relaxation times are then τ_ACC:V 1:1_ = 11 h, τ_ACC:V 1:2_ = 9 h, τ_ACC:V 1:3_ = 7 h and τ_ACC:V 0:1_ = 20 h.

### 3.3. Viscoelasticity of CaCO_3_ Pastes

Here, we study the time evolution of the elastic properties during the setting of the pastes. This evidences the existence of two distinct timescales: a short one of the order of the hour and a longer one that is consistent with the characteristic time of the phase transition. Finally, we interpret the rheological measurements using the colloidal gel model in the aim to provide insights on the paste microstructure and on the interaction between calcite grains.

#### 3.3.1. Measurement of Time Evolution of Viscoelastic Properties

**Figure 4:**
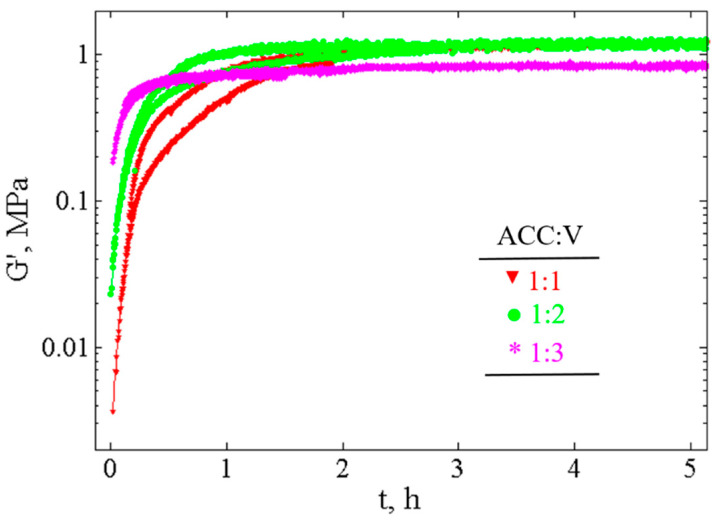
Time evolution of storage modulus, G′, for three different CaCO_3_ cement compositions (ACC:V 1:1, 1:2 and 1:3) measured at constant frequency, f = 10 Hz, and deformation, *γ* = 0.003%.

In order to map the strength evolution of the paste during recrystallization, we measured the viscoelastic properties with time. The storage modulus, *G*′, is typically one order of magnitude larger than the loss modulus, *G*″ (not shown), revealing the elastic-like behavior of the pastes. Figure 4 presents the evolution of the storage modulus, *G*′, over 5–6 h for three different CaCO_3_ cement pastes. For each composition, we show the fastest and the slowest recorded evolutions.

One observes that the initial rigidity of the pastes depends on the composition and that its value increases as the ACC:V ratio decreases. A smaller ACC:V ratio corresponds to a larger number of vaterite particles and smaller interparticle distances between vaterite particles. Since the interparticle forces are short range, the rigidity of the paste should increase as the interparticle distances decrease and consequently, as the ACC:V ratio decreases. This dependence has been reported previously not only for cement pastes but for several systems [34,35].

**Figure 5:**
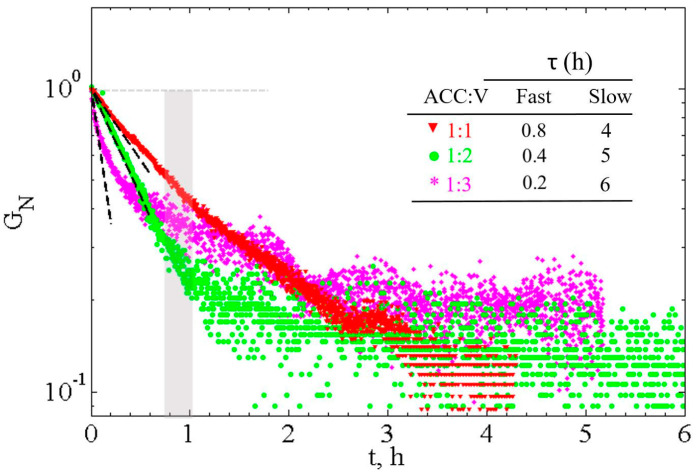
Time evolution of the normalized storage modulus, *G_N_*′, for three different CaCO_3_ cement compositions (ACC:V 1:1, 1:2 and 1:3) measured from the time evolution of the elastic modulus. The average characteristic reaction times, *τ_i_*, are also included.

For all studied pastes, the storage modulus, *G*′, increases progressively with time until it reaches a value of ~1 MPa, independent of the composition. Further insights from the data can be extracted by plotting the normalized final storage modulus, $G_{N}^{\prime },$ defined as:

(6) $$G_{N}^{\prime }=\frac{G^{\prime }\left(t_{f}\right)-G^{\prime }\left(t\right)}{G^{\prime }\left(t_{f}\right)-G^{\prime }\left(t_{0}\right)}$$

where $t_{0}$ is the initial time and $t_{f}$ is the final time (~6–10 h) as the plateau value is reached. This definition allows studying the time dependence of the storage modulus independently of its initial value and indicates the distance to the final value. Due to the subtraction ($G^{\prime }\left(t_{f}\right)-G^{\prime }\left(t\right)$), a decrease of $G_{N}^{\prime }$ corresponds to an increase of $G^{\prime }$. Figure 5 shows the time evolution of $G_{N}^{\prime }$ for the three different CaCO_3_ cement pastes examined here. One observes two distinct timescales; first, a short timescale $\tau_{1}^{\prime }$ (steep slope), then, a long timescale $\tau_{2}^{\prime }$ (small slope), that are determined by $\ln G_{N}^{\prime }=-t/\tau_{i}^{\prime }$. The calculated characteristic times averaged over all the measurements, $\tau_{1}^{\prime }$, are 0.8 ± 0.1 h, 0.4 ± 0.02 h and 0.2 ± 0.02 h, and $\tau_{2}^{\prime }$, are 4 ± 1 h, 5 ± 1 h and 6 ± 1 h for ACC:V 1:1, 1:2 and 1:3 compositions, respectively.

The rheological measurements evidence a fast increase of *G*′ with a timescale of typically 0.5 h followed by a slow increase of *G*′ with a timescale ~5 h. Then, *G*′ reaches a plateau of ~1 MPa, whose value is similar to *G*′ measured for calcite pastes with facetted grains of 70 nm size [28].

Note that the short characteristic time of typically 0.5 h evidenced by the rheological measurements is not detected by the phase transformation measurements. So, this time probably does not correspond to a phase transformation and has another origin that we will try to specify in the following section. As for the long characteristic time, it is of the same order of the one of the phase transformations. 

#### 3.3.2. Fast Increase of Elastic Modulus: Smoothing of Grains

The first fast strengthening of the paste is too slow to be due to merely reorganization of the grain packing. The rotational diffusion time of a vaterite grain, $\tau =\frac{4\pi \eta R^{3}}{k_{B}T}$, suggests reorganization on a characteristic timescale of less than a second. We will argue that smoothing of surfaces at grain contacts is the most likely controlling process. Indeed, the roughness of the contact is crucial for the interaction between particles and consequently for the macroscopic mechanical properties.

Recent molecular dynamics simulations of calcite–calcite interactions in water [36] show that there is an attractive energy minimum of −50 mJ·m^−2^ at a distance of 0.8 nm (4 layers of water) between perfectly smooth surfaces and an attractive well width of less than 0.2 nm. If the surfaces are not flat, one needs to convolute the interaction potential with the roughness [37], which results in no attractive interactions between calcite particles for roughness larger than 3 nm, a result that agrees well with other simulations and experiments on calcite [38,39]. As a result, we expect that smoothing of a contact induces an increase of the attraction between grains and consequently an increase of the storage modulus. We can also calculate the characteristic time of the smoothing process. 

**Figure 6:**
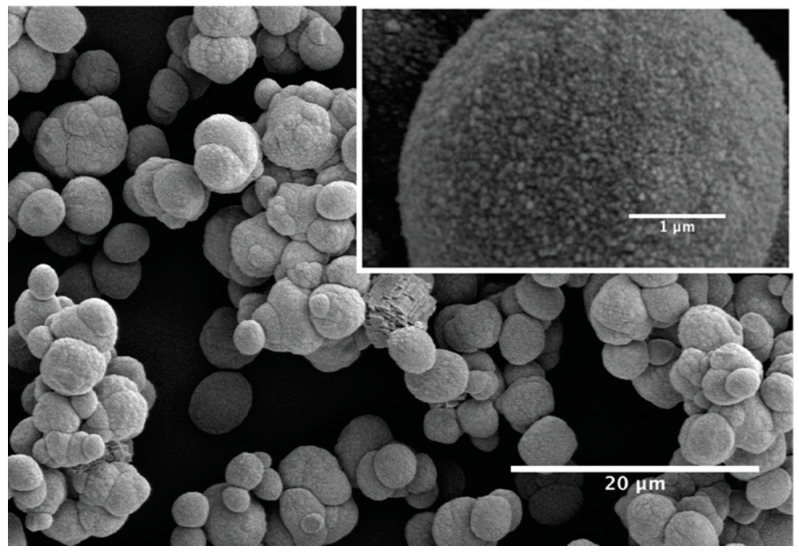
Vaterite at initial stage of phase transformation. Inset: Magnification of a vaterite grain demonstrating the surface roughness consisting of ~100 nm spheres on the 2–5 µm large vaterite grains.

As the ACC quickly dissolves, the mechanical properties of the paste are dominated by the interaction between vaterite grains, which depend on their shapes and interparticle distances. From SEM micrographs (see Figure 1B and Figure 6), vaterite particles can be viewed as spherical particles of characteristic radius *r_v_* = 2–5 µm, with surfaces consisting of 100 nm spheroids (see inset of Figure 6). The roughness of those spheroids can be estimated to typically ~10 nm. Such roughness is large compared to the range of attractive interaction, such as van der Waals, and thus initially causes a reduced attraction or a pure repulsive interaction between the vaterite grains, as shown previously [37,38]. A radius of curvature of the small vaterite spheres (*r_s_* = 50 nm) would cause an increase in the surface energy of the surface of ΔG=γVm(1rs−1rv)=150 J·mol^−1^, where, for the lack of vaterite surface energy, we have used the calcite surface energy. This chemical potential difference would drive local reordering and smoothing of the surface by recrystallization. Dissolution of the highest points will occur at a velocity of $v=V_{m}k_{d,v}{\left(e^{\frac{\Delta G}{RT}}-1\right)}^{0.86}$ [31]. Using *k_d,v_* of Cubillas et al. [31], *v* ~ 30 nm/h. If we assume that the dissolution of ~10 nm of the protruding spheres is the rate-limiting step, it would take ~0.3 h to smooth the rough vaterite surfaces and achieve attractive interactions between the vaterite particles. This would be the same order of magnitude as the fast timescale of the increase of the storage modulus, 0.2–0.8 h. This suggests that the fast increase of the elastic modulus is due to an increase of the interacting area and consequently, an increase of the attraction between grains.

#### 3.3.3. Structure of the Pastes

**Figure 7:**
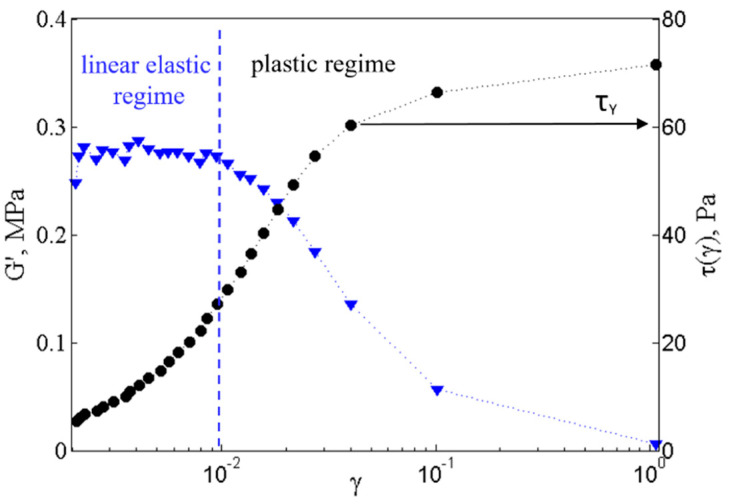
Rheological properties of 1:2 ACC:V suspension of 36% volume fraction. The shear modulus *G*′ (blue triangle down) and the shear stress *τ* (black circle) are plotted as a function of the shear strain *γ* measured through an amplitude sweep at f = 1 Hz. The vertical dashed line delineates the elastic and the plastic regimes. The horizontal plain line indicates the value of the yield stress defined as the value of the shear stress.

To go one step further and study the link between macroscopic properties (rheology) and microscopic properties (structure and interaction between grains), in this section we explore the structure of the initial paste (ACC:V). To do so, we study the influence of the volume fraction, denoted Φ, on the elastic modulus *G*′ and the yield stress yield $\tau_{Y}$. These rheological properties are obtained by applying an amplitude sweep, i.e., an increasing oscillating deformation, to the pastes for all studied mixing ratios (ACC:V 1:1; 1:2, 1:3) and volume fractions. An example of such amplitude sweep is shown in Figure 7: the elastic modulus and the shear stress are plotted as a function of the shear strain. The elastic modulus first exhibits a plateau at small strains corresponding to the elastic linear regime, followed by a drastic decrease corresponding to the plastic and flow regimes. On the other hand, the shear stress first increases linearly with strain in the elastic regime then saturates as the paste enters in the plastic regime. Here, the yield stress, $\tau_{Y},$ is defined as the value of the shear stress at the beginning of the stress plateau reached for shear strain γ ~ 5.10^−2^–10^−1^%.

**Figure 8:**
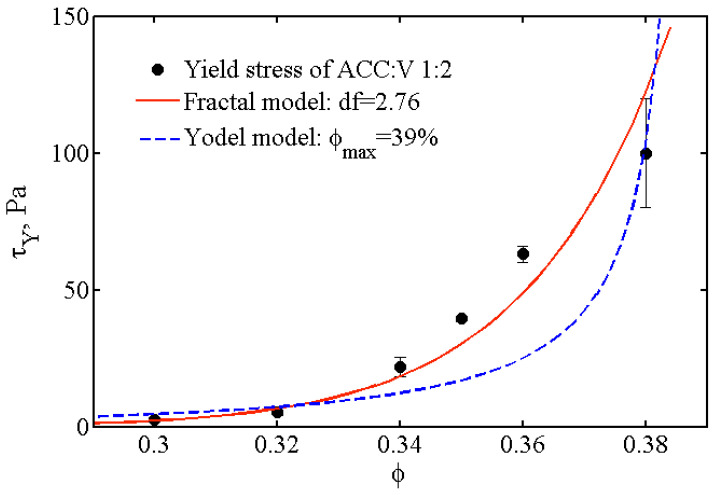
Dependence of the yield stress of ACC:V (1:2) paste with the volume fraction. Comparison of the experimental data with the Yodel model [22] (blue curve) and the fractal model of Shi et al. [24] (red curve).

Note that these measurements are performed just after preparation of the paste, i.e., typically 10–15 min after mixing, and last for ~10 min. A drawback of this protocol is that the paste properties change during the measurement, but it allows us to estimate the volume fraction range for which the initial suspension is a paste. More precisely, we manage to measure an elastic modulus and a yield stress for 20% ≤ f ≤ 38%. This volume fraction range suggests [40] that flocculation occurs between grains. Indeed, the volume fraction for which the yield stress diverges, f*_div_*, is much smaller than the maximum packing fraction of identical hard spheres (64%). Each floc is composed by aggregated particles and water, which explains the smaller value of the maximal volume fraction. To go one step further, we analyzed the volume fraction dependence of the yield stress, *τ_Y_*(f), in terms of Yodel’s model [22] and of Shi’s model [23,24], both developed for suspension of flocculated colloids (see Figure 8). 

In short, Yodel’s model consists in counting the interparticle bonds that cannot be broken and that are responsible for the yield stress. It takes into account the interparticle forces such as van de Waals or DLVO (Derjaguin-Landau-Verwey-Overbeek), the particle size, the maximum packing and the percolation threshold. For high volume fraction (f>25%), the percolation threshold can be neglected and the expected dependence of the yield stress with the volume fraction is expected to be:

(7) $$\tau_{Y}≅m\frac{f^{3}}{{\;f}_{div}\left({\;f}_{div}-\;f\right)}$$

where *m* depends on the particle size and on the interaction between particles. The comparison between the Yodel model and our experimental data (see Figure 8) for ACC:V (1:2) paste suggests a maximum packing of 39%. However, the adjustment of the experimental data by this model is not very good, in particular the divergence of the yield stress with the volume fraction is not well taken into account.

We now turn to the Shi’s model [23,24], a scaling theory that considers the colloidal gels as a dense suspension of flocs which are fractal objects composed by flocculated colloids. In this model, the rheological properties are set by the weakest link between particles or flocs. At high volume fractions, corresponding to our study, the weakest link is between flocs (named weak-link regime) and the yield stress is expected to depend on the volume fraction as a power law:

(8) $$\tau_{Y}\sim mf^{p}$$

where *m* depends on the interparticle forces and the particle size and the exponent p depends on the fractal dimension, *d_f_*, of the flocs. More precisely, $p=\frac{d+x}{d-d_{f}}$, where d is the Euclidean dimension and *x* is the backbone of the clusters, often taken equal to 1 [28].

The adjustment of our data with the power law is quite satisfactory and suggests a fractal dimension of the flocs: *d_f_* ~ 2.76.

In summary, this analysis suggests that our pastes can be considered as a dense suspension of flocs composed by aggregated colloids whose rheological properties depend of the interparticle force, particle size, surface roughness and contact area between particles. Any process modifying one of these parameters automatically induces a change of the rheological properties (elastic modulus and yield stress). 

#### 3.3.4. Discussion of Final Hardening of Cement

The plateau value of *G*′ ~ 1 MPa, reached after a few hours of aging, is very far from the shear modulus of the hardened cement of ~1 GPa and of a pure calcite of 35 GPa. This signifies that here, the elasticity of the paste is due to weak interactions inside flocs and between branched flocs of calcite [28]. 

The final strength of the cement is attained with a different timescale at a later stage after the experiments presented here. We will, however, make some reflections on the relation to the present results. During drying, the main structure is locked and does not change appreciably, except for loose grains that are pulled towards the closest neighbors by capillary forces as menisci are moving during drying. Stiff grain contacts with stronger, covalent bonds are formed where weaker van der Waals (vdW) forces dominated the wet paste. The elastic modulus increases by 3 orders of magnitude, probably mainly due to the shear strength of covalent forces as opposed to the lack of shear strength in wet grain contacts with vdW forces. The relatively low yield stress of ~1 MPa [41], however, suggests that the open structure inherited from the paste results in relatively few and small covalently bounded grain contacts. This suggests that any attempt to develop calcite cements with a higher yield strength should target the recrystallization period studied here to achieve a more compact structure with larger grain contacts.

## 4. Conclusions

Calcium carbonate cements have been synthesized by mixing amorphous calcium carbonate and vaterite powders with water to a cement paste to study how mechanical strength is created during the setting reaction. Two complementary tools have been used: In-situ XRD was used to monitor the phase transformation of ACC and vaterite phases into calcite and a rotational rheometer was used to monitor the strength evolution. XRD gives access to the kinetics of the phase transformations that could not be understood through rheological experiments or from previous results in fluid reactors. Viscoelastic measurements are more sensitive to interparticle forces and to the grain morphology and give access to the kinetics of grain smoothening, not detected by XRD. The combination of the two techniques allows to link microstructure to macroscopic properties.

There are two characteristic timescales of the strengthening of the cement paste. The short timescale of the order 1 h is shown to be controlled by smoothening of the vaterite grains, allowing closer and therefore adhesive contacts between the grains. The long timescale of the order 10–50 h, observed both in rheological and XRD measurements, is controlled by the phase transformation of vaterite into calcite. The new results of phase transformations have been analyzed using a dissolution–diffusion–precipitation model that reveals that the phase transformation in a paste is limited mainly by diffusion in the liquid phase and partially by vaterite dissolution. This is contrary to previous results in fluid reactors, where calcite growth is the rate-limiting step.

We also found that the evolution of shear strength with solid volume fraction is best explained by a fractal model of the paste structure and that the Yodel model does not fit our data.

The final conclusion from this study is that at short times (~1 h), contact areas and attractive forces between grains increase due to grain smoothing and the phase transformation exchanges the strength bearing phase from vaterite into calcite. This exchange occurs at 10–50 h in dense suspensions.

## Figures and Tables

**Table 1:** Cement mixture nomenclatures, weight ratios of solid precursors and water/solid ratio used for each test.

	Weight Ratio (%)		Water/Solid Ratio	
Mixture Nomenclature	ACC ^1^	V ^2^	For XRD Tests	For Rheological Tests
ACC:V 1:0	100	0	0.43	(not tested)
ACC:V 1:1	50	50	0.43	0.65
ACC:V 1:2	33	66	0.43	0.65
ACC:V 1:3	25	75	0.43	0.65
ACC:V 0:1	0	100	0.43	(not tested)

^1^ ACC—Amorphous Calcium Carbonate; ^2^ V—Vaterite.

## Supplementary Materials

The following are available online at https://www.mdpi.com/1996-1944/13/16/3582/s1, Supplementary text and Figure S1: Local slopes of calcite mass fractions, Figure S2: Normalized mass evolution.
