# Supplementary material for: Mechanisms of Phase Transformation and Creating Mechanical Strength in a Sustainable Calcium Carbonate Cement
Source: Materials (Basel). 2020 Aug 13;13(16):3582. doi: 10.3390/ma13163582 (PMC7476014; doi:10.3390/ma13163582)
Supplement: Supplementary file 1 [file materials-13-03582-s001.pdf]

materials

Supplementary material to:

Mechanisms of Phase Transformation and Creating Mechanical Strength in a Sustainable Calcium Carbonate Cement

Jesús Rodríguez-Sánchez [1,2], Teresa Liberto [3,4], Catherine Barentin [4,5] and Dag Kristian Dysthe [1,*]

[1] Physics of Geological Processes (PGP), The NJORD Centre, Department of Physics, University of Oslo, P.O. Box 1048 Blindern, Oslo, Norway; j.rodriguez-sanchez@sheffield.ac.uk
[2] Department of Materials Science and Engineering, University of Sheffield, S10 2TN Sheffield, UK;
[3] Building Physics and Construction Ecology, Faculty of Civil Engineering, Institute of Materials Technology, Vienna University of Technology, 1030 Vienna, Austria; teresa.liberto@tuwien.ac.at
[4] Institut Lumière Matière, Université Claude Bernard Lyon 1, CNRS, F-69622 Villeurbanne, France; catherine.barentin@univ-lyon1.fr
[5] Institut Universitaire de France, 75231 Paris, France
* Correspondence: d.k.dysthe@fys.uio.no

Received: 22 July 2020; Accepted: 11 August 2020; Published: date

S1. Growth and Dissolution Rates

S.1.1 Growth or dissolution control

The crystal growth/dissolution rate, *r*, (dissolution if *r* is negative) can be expressed as:

$$r = A^{-1}\frac{\mathrm{d}m}{\mathrm{d}t} = \pm\, k \cdot e^{\frac{-E_a}{RT}} \cdot f(\Delta G)$$

where k is the growth rate constant (kg s^{-1} m^{-2}), A is the reactive surface area of the growing phase, dm/dt is the change in solid mass of the phase per time, E_a is the apparent activation energy of the overall reaction, R is the gas constant, T is the absolute temperature and $f(\Delta G)$ introduces the dependence of the overall growth rate on the supersaturation state of the system expressed as a function, f, of the Gibbs free-energy change for the growth reaction, ΔG. The velocity of the growing/dissolving surface is

$$v = V_m r,$$

where V_m is the molar volume (vaterite: $3.8 \cdot 10^{-5}$ $m^3 \cdot mol^{-1}$, calcite: $3.7 \cdot 10^{-5}$ $m^3 \cdot mol^{-1}$).

The ΔG function may have a variety of forms. However, since the overall reaction here presented is an elementary reaction, $f(\Delta G)$ can be derived from transition state theory (TST) [1,2] in the following way:

$$f(\Delta G) = \left(e^{\frac{\Delta G}{RT}} - 1\right)^n \approx \left(\frac{\Delta G}{RT}\right)^n$$

The equation of the phase evolution rate when transport is much faster than growth and dissolution is

$$\frac{\mathrm{d}m_c}{\mathrm{d}t} = r_c A_c - r_v A_v$$

If the vaterite grains are of similar size there will be approximately a constant number of vaterite grains the whole time and therefore $A_v \propto m_v^{2/3}$. If all calcite nuclei are formed initially and the number of calcite grains remain constant, then $A_c \propto m_c^{2/3}$.

If the rate of calcite growth is much slower than the rate of vaterite dissolution, the aqueous $CaCO_3$ concentration will equal the vaterite solubility and the driving force for the growth will be kept constant at the difference ΔG of the vaterite and calcite phase. Inversely, if vaterite dissolution is much slower than calcite growth, the aqueous $CaCO_3$ concentration will equal the calcite solubility.

Thus, the same ΔG will be the constant driving force of vaterite dissolution. Since everything is constant but the mass and the area of the growing phases the rate equation simplifies to

$$\frac{\mathrm{d}m_c}{\mathrm{d}t} = r'_c\, m_c^{2/3} \;\Rightarrow\; \frac{m_c}{m_{c,0}} = r'_c \left(\frac{t}{t_f}\right)^3$$

for calcite growth control and

$$\frac{\mathrm{d}m_v}{\mathrm{d}t} = -|r'_v|\, m_v^{2/3} \;\Rightarrow\; \frac{m_v}{m_{v,0}} = r'_v \left(1 - \frac{t}{t_f}\right)^3,$$

for vaterite dissolution control. The final time is inversely proportional to the rate: $t_f \propto r'^{-1}_v$, Since $m_{vf} \rightarrow 0$ the vaterite dissolution control model can be written:

$$\frac{m_v(t) - m_{v0}}{m_{vf} - m_{v0}} \propto 1 - \left(1 - \frac{t}{t_f}\right)^{\alpha}.$$

Previous phase transformation studies in batch reactors with well stirred liquid solutions reported by Ogino et al. [3] and Rodriguez-Blanco et al. [4] found that $m_c \propto \left({}^{t}/_{t_f}\right)^3$ and consequently concluded that the phase transformation ACC - vaterite - calcite was controlled by the rate of calcite growth.

S.1.2 Nucleation Control

Even though the calcite growth rate is 2-10 times larger than the vaterite dissolution rate (see below), a restricted number of calcite nuclei may render the phase transformation controlled by calcite growth. If N0 new nuclei are formed initially and no new nuclei are formed after that, the mass of calcite will grow as $m_c = r''_c t^3$, where $r''_c \propto N_0$ (assuming constant supersaturation as above). This means that even though the rate constant of calcite growth is larger than that of vaterite dissolution the effective growth rate is proportional to the number of nuclei and can be much smaller than the vaterite dissolution rate. The observation by Ogino et al. [3] and Rodriguez-Blanco et al. [4] found that $m_c \propto \left({}^{t}/_{t_f}\right)^3$ is thus probably due to the limited number of calcite nuclei in the well stirred liquid solutions.

S.1.3 Diffusion Control

If, on the other hand, diffusion is much slower than the growth and dissolution rates the transformation rate will be $\frac{dm_c}{Vdt} = -D\Delta c\, l_D^{-2}$, where l_D is the diffusion distance, D the diffusion coefficient, V a standard volume, $\Delta c = c_{sol,V} - c_{sol,C}$ and $c_{sol,V}$ and $c_{sol,C}$ are the solubilities of vaterite and calcite. Different models of how the diffusion distance l_D evolves will yield different time dependence. We will assume that all calcite grains nucleate close to vaterite grains so that initially the diffusion distance is small. As the calcite grains grow the diffusion distance is proportional to the radius of the calcite grains, thus $l_D \propto m_c^{1/3}$. This yields the rate equation:

$$\frac{\mathrm{d}m_c}{\mathrm{d}t} \propto m_c^{-2/3} \;\Rightarrow\; m_c \propto \left(\frac{t}{t_f}\right)^{3/5}$$

This simple model is captures an essential part of the phase transformation of the cement. A numerical simulation is probably necessary to describe the full complexity.

S.1.4 Driving Forces for Dissolution

Curvature: $\Delta G = \gamma V_m / r$,

where r is the radius of curvature of the surface $\gamma = 0.2$ J m^{-2} is the surface energy [5].

Phase change:

ACC - Calcite: $\Delta G = 15$ kJ mol^{-1} [6]

Vaterite - Calcite: $\Delta G = 6.2$ kJ mol^{-1} [7]

S.1.5 Rate Constants

Dissolution rate of vaterite at 20°C:

$r_{d,v}$=-$k_{d,v}\left(e^{\frac{\Delta G}{RT}}-1\right)^{0.86}$, and $k_{d,v}=2.69\cdot 10^{-6}$ mol·m^{-2}s^{-1} from Cubillas et al [8] . Thus, for a system controlled by the energy difference of vaterite and calcite, $\Delta G=6.2$ kJ mol^{-1}, $r_{d,v}$=-2.2·10^{-5} mol·m^{-2}s^{-1}.

Growth rate of calcite at 20°C:

$r_{g,c}$=$k_{g,v}\left(e^{\frac{\Delta G}{RT}}-1\right)$, where $k_{g,c}=3160$ nm h^{-1} = 2.37·10^{-5} mol·m^{-2}s^{-1} from single crystal experiments [9] and $k_{g,c}$ = 4.6·10^{-6} mol·m^{-2}s^{-1} from batch reactor experiments [10]. Thus for a system controlled by the energy difference of vaterite and calcite, $\Delta G=6.2$ kJ mol^{-1}, $r_{d,v}$ is in the range from 5.4·10^{-5} to 2.8·10^{-4} mol·m^{-2}s^{-1}.

Mean diffusion coefficient of $CaCO_3$ ions: $D=1.1\cdot 10^{-9}$ m^2 s^{-1}.

S.2 Analysis of Phase Transformation Data

The first step in our analysis is to estimate the final time, t_f. From Figure S2 one observes that there is some vaterite left in the cement after the phase transformation has slowed down to a rate that is essentially zero at the resolution of our experiments. As a definition of t_f we require that the local slope of the mass fraction curves is smaller than the fluctuation in the slope at t_f. This is done using a local regression using weighted linear least squares and a 1st degree polynomial model with a window of 15 data points. Figure S1 shows the slopes of the calcite mass fractions around t/t_f=1.

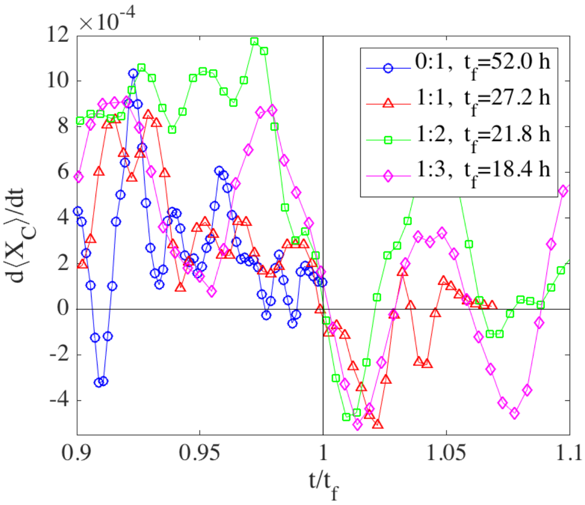

Figure S1. Local slopes of calcite mass fractions at times approaching t_f.

Assuming dissolution, growth or diffusion to be the rate limiting step of the phase transformation yields models with different power law dependencies. There are two ways one may rescale the data to compare to the models. Either by rescaling the mass and by the final time, t_f:

$$\frac{m(t)-m_0}{m_f-m_0}\propto\left(\frac{t}{t_f}\right)^{\alpha}$$

or by shifting the starting time, t_s, and rescaling by the final time, t_f:

$$1-X_v\propto X_C\propto\left(\frac{t_s+t}{t_f}\right)^{\alpha}$$

In Figure S2 we have used both ways of rescaling and compared to the models that yield different exponents α.

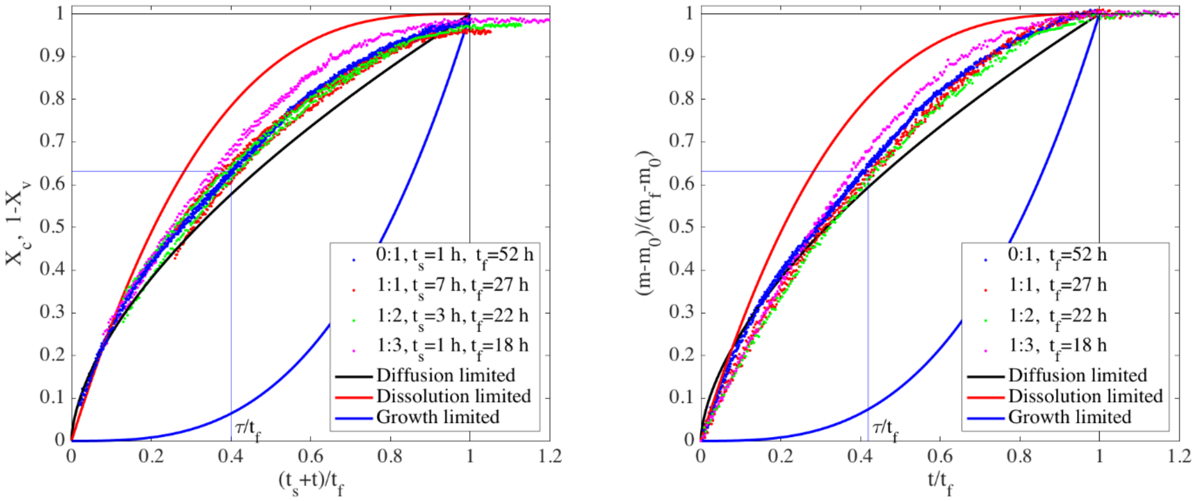

Figure S2. Normalized mass evolution with normalized time for four different $CaCO_3$ cement compositions (0:1, 1:1, 1:2 and 1:3 wt.% ACC:V). Using a standard definition of the relaxation time τ: $\ln[(m_0-m)/(m_f-m_0)] = t/\tau$, the relaxation times are $\tau \sim 0.4\ t_f$, thus $\tau = 21$, 11, 9 and 7 hours, respectively. The solid lines demonstrate the three different phase transformation models.

The time constants, t_f, are 3-10 times longer than those found for stirred liquid experiments with ACC:V=0:1, $t_{f,liquid}$ =5 h at 20°C [4]. One observes that the two ways of rescaling the data is roughly equivalent within the accuracy of the data. Using a standard definition of the relaxation time ◎: $\ln[(m_0-m)/(m_f-m_0)] = \ln(X) = t$ /◎, the relaxation times τ, are $\tau_{ACC:V\ 1:1} = 11$ h, $\tau_{ACC:V\ 1:2} = 9$ h, $\tau_{ACC:V\ 1:3} = 7$ h and $\tau_{ACC:V\ 0:1} = 21$ h.

Supplementary References

1. Lasaga, A.C.: Kinetics of Geochemical Processes, in: Lasaga, A.C., Kirkpatrick, R.J. (Eds.); Mineralogical Society of America: Urbana, Illinois, USA, 1981; 8, p. 81.
2. Aagaard, P.; Helgeson, H.C. Thermodynamic and kinetic constraints on reaction rates among minerals and aqueous solutions. I. Theoretical considerations. *Am. J. Sci.* **1982**, *282*, 237–285. doi:10.2475/ajs.282.3.237.
3. Ogino, T.; Suzuki, T.; Sawada, K. The formation and transformation mechanism of calcium carbonate in water *Geochim. Cosmochim. Acta.* **1987**, *51*, 2757–2767. doi:10.1016/0016-7037(87)90155-4.
4. Rodriguez-Blanco, J.D.; Shaw, S.; Benning, L.G. The kinetics and mechanisms of amorphous calcium carbonate (ACC) crystallization to calcite, via vaterite *Nanoscale.* **2011**, *3*, 265–271. doi:10.1039/c0nr00589d.
5. Royne, A.; Bisschop, J.; Dysthe, D.K. Experimental investigation of surface energy and subcritical crack growth in calcite *J. Geophys. Res. Solid Earth.* **2011**, *116*, 1–10. doi:10.1029/2010JB008033.
6. Wolf, G.; Günther, C.Thermophysical investigations of the polymorphous phases of calcium carbonate *J. Therm. Anal. Calorim.* **2001**, *65*, 687–698. doi:10.1023/A:1011991124181.
7. Plummer, L.N.; Busenberg, E. The solubilities of calcite, aragonite and vaterite in CO-H_2O solutions between 0 and 9OC, and an evaluation of the aqueous model for the system $CaC0_3$-CO_2-H_20 *Gcochlmica Cosmochim. Acta.* **1982**, *46*, 1011–1040.
8. Cubillas, P.; Köhler, S.; Prieto, M.; Chaïrat, C.; Oelkers, E.H. Experimental determination of the dissolution rates of calcite, aragonite, and bivalves *Chem. Geol*. **2005,** *216*, 59–77. doi:10.1016/j.chemgeo.2004.11.009.
9. Li, L.; Sanchez, J.R.; Kohler, F.; Røyne, A.; Dysthe, D.K. Microfluidic Control of Nucleation and Growth of $CaCO_3$ *Cryst. Growth Des.* **2018**, *18*, 4528–4535. doi:10.1021/acs.cgd.8b00508.
10. Reddy, M.M.; Plummer, L.N; Busenberg, E. Crystal growth of calcite from calcium bicarbonate solutions at constant $PCO_2$and 25°C: a test of a calcite dissolution model *Geochim. Cosmochim. Acta.* **1981,** *45*, 1281–1289. doi:10.1016/0016-7037(81)90222-2.

© 2020 by the authors. Submitted for possible open access publication under the terms and conditions of the Creative Commons Attribution (CC BY) license (http://creativecommons.org/licenses/by/4.0/).
